# Intraarticular location predicts cartilage filling and subchondral bone changes in a chondral defect: A randomized, blind, long-term follow-up trial involving 82 rabbit knees

**DOI:** 10.3109/17453674.2010.524593

**Published:** 2010-10-08

**Authors:** Stig Heir, Asbjørn Årøen, Sverre Løken, Steinar Sulheim, Lars Engebretsen, Finn P Reinholt

**Affiliations:** ^1^Martina Hansens Hospital, Bærum; ^2^Institute of Surgical Research, Oslo University Hospital Rikshospitalet, Oslo; ^3^Oslo Sports Trauma Research Center, Norwegian School of Sport Sciences, Oslo; ^4^Orthopedic Center, Oslo University Hospital Ullevål, Oslo, and Faculty of Medicine, University of Oslo; ^5^Innlandet Hospital Lillehammer, Lillehammer; ^6^Division of Pathology, University of Oslo, and Oslo University Hospital Rikshospitalet, Oslo, Norway

## Abstract

**Background and purpose:**

The natural history of, and predictive factors for outcome of cartilage restoration in chondral defects are poorly understood. We investigated the natural history of cartilage filling subchondral bone changes, comparing defects at two locations in the rabbit knee.

**Animals and methods:**

In New Zealand rabbits aged 22 weeks, a 4-mm pure chondral defect (ICRS grade 3b) was created in the patella of one knee and in the medial femoral condyle of the other. A stereo microscope was used to optimize the preparation of the defects. The animals were killed 12, 24, and 36 weeks after surgery. Defect filling and the density of subchondral mineralized tissue was estimated using Analysis Pro software on micrographed histological sections.

**Results:**

The mean filling of the patellar defects was more than twice that of the medial femoral condylar defects at 24 and 36 weeks of follow-up. There was a statistically significant increase in filling from 24 to 36 weeks after surgery at both locations.

The density of subchondral mineralized tissue beneath the defects subsided with time in the patellas, in contrast to the density in the medial femoral condyles, which remained unchanged.

**Interpretation:**

The intraarticular location is a predictive factor for spontaneous filling and subchondral bone changes of chondral defects corresponding to ICRS grade 3b. Disregarding location, the spontaneous filling increased with long-term follow-up. This should be considered when evaluating aspects of cartilage restoration.

Focal articular cartilage injuries of the knee are common (Hjelle et al. 2002, Aroen et al. 2004) and they can impair patients' quality of life as much as severe osteoarthritis (Heir et al. 2010). The literature concerning the natural history of focal cartilage defects in patients, and the intrinsic factors affecting it, is limited (Linden 1977, Messner and Gillquist 1996, Drogset and Grontvedt 2002, Shelbourne et al. 2003, Loken et al. 2010). In experimental studies evaluating cartilage restoration in general, the importance of intrinsic factors such as the depth and size of the lesion and the time from when the lesion was made to evaluation have been emphasized (Shapiro et al. 1993, Hunziker 1999, Lietman et al. 2002). Which part of the joint is affected and whether or not the defect is weight-bearing are also of interest (Hurtig 1988, Frisbie et al. 1999). Most of these studies have, however, concerned defects penetrating the subchondral mineralized tissues corresponding to ICRS grade 4 (Brittberg and Winalski 2003). Access to bone marrow elements in these defects might be one of the strongest predictive factors for filling of the defect, making the importance of other factors difficult to evaluate (Hunziker 1999).

In experimental studies on pure chondral defects that do not penetrate the subchondral mineralized tissues, corresponding to ICRS grade 3b (Brittberg and Winalski 2003), the type of animal studied, the size of the lesion, and the location of the defects vary, and there is limited data on the influence of these parameters on outcome (Breinan et al. 2000). The information on spontaneous filling comes mainly from observations of untreated defects serving as controls (Grande et al. 1989, Brittberg et al. 1996, Breinan et al. 1997, 2000, Frisbie et al. 1999, 2003, Dorotka et al. 2005) and the information on subchondral bone changes is even more limited (Breinan et al. 1997, Frisbie et al. 1999). Although most human focal cartilage lesions are located on the medial femur condyle (Aroen et al. 2004), there have been few experimental studies involving untreated ICRS grade 3b defects on the medial femur condyle (Dorotka et al. 2005). According to a PubMed search, the rabbit knee is the most widely used experimental animal model for cartilage restoration (Årøen 2005). The locations of ICRS grade 3 chondral defects in the rabbit knee evaluated for spontaneous changes have included the patella (Grande et al. 1989, Brittberg et al. 1996) and, in one study, defects at the distal surface of the femur (Mitchell and Shepard 1976). The latter report did not, however, include quantitative data.

To our knowledge, the influence of the intraarticular location on the outcome of cartilage restoration and subchondral bone changes has not been thoroughly studied. Thus, the main purpose of our study was to test the hypothesis that the intraarticular location influences the spontaneous filling of a chondral defect that does not penetrate the subchondral bone. Secondly, we wanted to evaluate whether the intraarticular location would influence changes in the subchondral bone and degenerative changes as evaluated from macroscopic appearance and proteoglycan content of synovial fluid (Messner et al. 1993a).

## Methods

We used our established experimental animal model (Aroen et al. 2005). Adult New Zealand rabbits were included in a randomized study where circular lesions 4 mm in diameter were created in the patella of one knee and compared in pairwise fashion to identical lesions in the medial femoral condyle of the contralateral knee. The lesions were pure chondral—down to, but not penetrating the calcified layer—corresponding to ICRS grade 3b. Follow-up was 12, 24, and 36 weeks after initial surgery.

The main endpoint was difference in degree of tissue filling between the defects in the patella and the condyles at each follow-up. The secondary endpoints were difference in changes in density of subchondral mineralized tissue with time and difference in degenerative changes, evaluated by macroscopic appearance and joint fluid proteoglycan content, between the two locations at each follow-up.

### Care of animals

**Figure 1.**
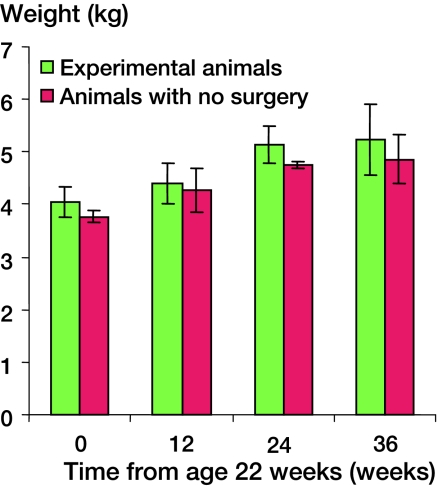
The experimental animals gained weight throughout the experimental period similar to that of the control animals. The number of experimental animals at time zero (age 22 weeks) equalled the total number of animals evaluated at the follow-ups (37), since they were all measured preoperatively. The numbers of experimental animals at 12, 24, and 36 weeks were 8, 11, and 18, respectively. The numbers of animals without surgery at time 0, 12, 24, and 36 weeks were 3, 3, 2, and 2, respectively.

The conditions for the animals, their diet, the use of sterile conditions perioperatively, capture of synovial fluid for proteoglycan analyses, anesthesia, analgesia, and the killing procedure have all been described previously (Aroen et al. 2005). Throughout the follow-up period, the experimental animals gained weight and achieved a weight at follow-up that was similar to that of control animals of corresponding age (Figure 1). The animals were allowed to move freely in their cages, and all of them were able to bear weight on both extremities immediately after surgery. The surgeons were experienced orthopedic surgeons and they were certified according to the rules of animal care and experimental surgery. The study was performed according to the guidelines for animal research at the University of Oslo and it was approved by the Committee for Experimental Animal Care of the Norwegian government.

### Experimental groups

41 adult New Zealand rabbits were used. The animals had defects created in both knees at the age of 22 weeks, in the patella of one and in the medial femoral condyle of the other. To avoid the possibility of the “learning curve” being a form of bias, the animals were block-randomized for killing at the follow-up time points. 12 animals were planned to be killed at 12 weeks, 12 at 24 weeks, and 17 at 36 weeks. However, due to substantial loss of animals during follow-up, the decision was made to spare animals to ensure that there was a sufficient number at 36 weeks of follow-up, since this was considered to be the most important follow-up time point of the study. Partly because of this shift in group sizes and partly because of other complications during the study, the final numbers of patellas and condyles available for evaluation were 8 and 8, respectively, at 12 weeks, 9 and 9 at 24 weeks, and 17 and 18 at 36 weeks of follow-up. The numbers of animals available for paired analysis (with sections from both knees available for evaluation) were 8 at 12 weeks, 7 at 24 weeks, and 17 at 36 weeks.

In addition, 20 knees from 10 animals with no initial surgery were evaluated by the same methods as those used for the experimental knees. The mean value for the 2 knees of each animal was used for calculations. 3 animals were killed at the age of 22 weeks, 3 at age 34 weeks (22 + 12), 2 at 46 weeks (22 + 24), and 2 at 58 weeks (22 + 36). The intention was to add these knees to a larger number of control specimens, harvesting the patella as a control specimen from knees with condylar defects and vice versa. However, since surgery had been performed on another joint surface within the same knee, the latter control specimens were excluded. Thus, due to the low number of “control” specimens, the data extracted were used for observational purposes only and were not included in statistical comparisons.

### Surgical technique

**Figure 2.**
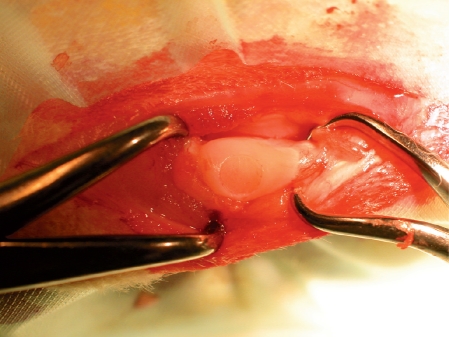

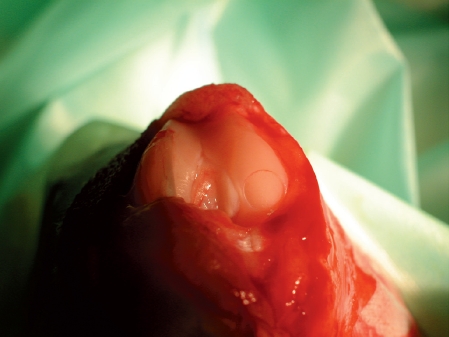
A. The 4-mm chondral defect in the rabbit patella. B. The 4-mm chondral defect in the rabbit MFC.

**Figure 3.**
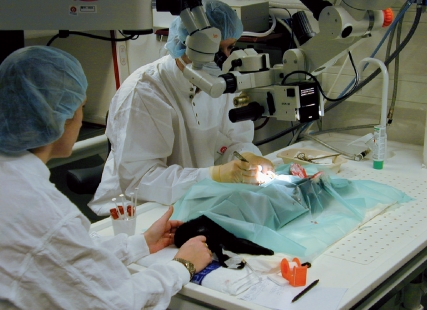
The set-up for surgery. A stereomicroscope ensured the complete removal of all cartilage above the tidemark without harming underlying tissue.

Through a medial parapatellar incision, a defect with a diameter of 4 mm was created in the patella of one knee (randomized to left or right) and in the medial femoral condyle of the contralateral knee, as previously described (Aroen et al. 2005) (Figure 2). A stereo microscope and small instruments were used (Figure 3), and care was taken to avoid any damage to the rims of the defects or to the underlying calcified cartilage. The defects were left untreated, the joint was irrigated, hemostasis was achieved, and wound closure was performed as previously described (Aroen et al. 2005).

### Killing of animals, macroscopic evaluation, and preparation for histological analysis

The animals were killed and synovial fluid was obtained as previously described (Aroen et al. 2005). The patellas and the femoral condyles were dissected free and gross morphological grading was performed. Changes to the cartilage corresponding to ICRS grade 1–2 and/or small osteophytes were categorized as minor changes, whereas changes exceeding that were categorized as major. The specimens were immersed in phosphate-buffered 4% paraformaldehyde for 1 week and decalcified in 20% formic acid until the bone was soft enough for sectioning. Cubes, measuring 8 × 8 mm and containing the defect at one side, were harvested from the specimens, dehydrated in graded alcohol and embedded in an epoxy resin. The patellas and condyles with no defects were handled in the same manner as the experimental specimens. To define the region for histological analysis, an area corresponding to the location of the experimental defects was outlined using the same kind of 4-mm biopsy punch as used when creating the defects in the experimental knees.

### Histology

**Figure 4.**
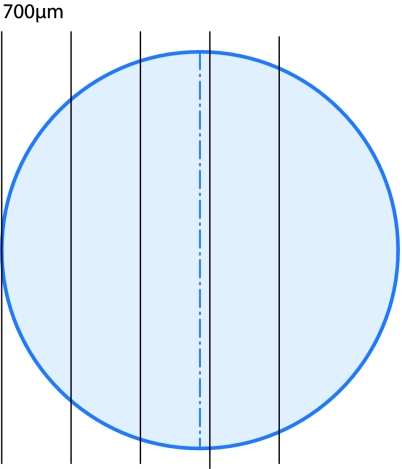
Starting at the rim of the defect, 5 levels of sectioning, each 700 μm further into the 4-mm defect, ensured that a central section would be analyzed.

The cubes were sectioned from one longitudinal surface, with random orientation of the defect (anterior-posterior or medial-lateral) (Gundersen et al. 1988). From the point at which the rim of the defect was reached, sections—each 1–2 μm thick—were captured at 5 levels, each level 700 μm further into the defect. This technique ensured that one of the sections would represent a level at a maximum distance of 350 μm from the very center of the defect (Figure 4). The sections were stained with tolouidin blue and photographed at 40× magnification using a digital camera mounted on the microscope. An interactive semiautomatic image analysis program (Analysis Pro; Olympus Soft Imaging Solutions, Münster, Germany) was used for all measurements. The section closest to the center of each defect (largest diameter) was included in the statistical analyses. The observer was blind as to the location of the defects.

### Estimation of tissue filling

**Figure 5.**
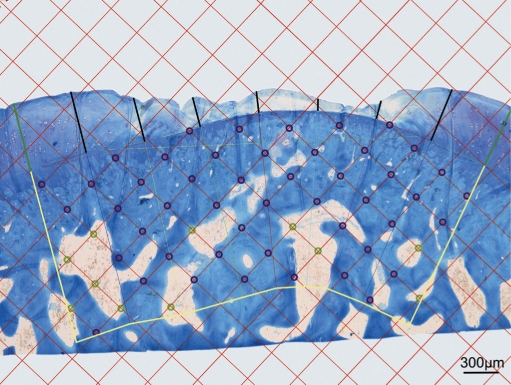
Histological section of a defect in the patella stained with tolouidine blue and photographed at 40× magnification. The section has been modified, highlighting some of the graphics superimposed by the software Analysis Pro. The two green lines outline the height of the shoulders measured. The seven black lines indicate the height of the tissue filling the defect. The yellow lines together with the tidemark frame the area of interest for subchondral mineralized tissue evaluation. The intersections of the red grid are marked with blue circles when overlying mineralized tissue, and with green circles when not.

The borders of the defects were identified by the interfaces between presumed original cartilage and newly formed fibrocartilaginous/fibrous repair tissue on both sides. The midpoint along the tidemark of the defect was defined. From the midpoint, sectors 0.5 mm in length were marked along the tidemark to each side until 2 mm from midpoint was reached on each side, representing the tidemark at the base of the “shoulders” of the 4-mm defect. Cartilage height was measured at these 2 shoulder points, as was tissue height at the 7 intersection points of the 0.5-mm sectors (Figure 5). Tissue filling was estimated by relating the mean height of tissue at the 7 defined points to the mean cartilage height of the 2 shoulders of the same defect, expressed as percentage filling. Whenever one of the shoulders was not measurable technically, the one shoulder that was left served as the reference. This occurred in sections from 1 patellar defect and 1 condylar defect.

### Estimation of the density of mineralized subchondral tissue

The density of the mineralized subchondral tissue was estimated in a region immediately beneath the condral defect. The depth of the region was 1.5 mm measured from the tidemark, thus the shape of the region depended on the curvature of the tidemark (Figure 5). The morphometry was performed by point counting (Gundersen et al. 1988) using computer software. A grid with 0.3 mm between test lines was superimposed on the micrograph. The intersections between the test lines served as test points. Subchondral mineralized tissue density was expressed as the number of test points overlying mineralized tissue relative to the total number of test points within the region of interest. The counting was repeated twice for each defect with the orientations of the grid randomly selected on both occasions. The mean value of the 2 measurements was used for statistical analysis. A similar technique was used by Løken et al. (2008), demonstrating a variance of less than 10% between measurements and between observer.

### Analysis of synovial fluid

41 animals had initial surgery at 22 weeks of age. From 60 of the knees, a wash-out sample of the synovial fluid containing a minimum of 0.75 mL collected before surgery (time zero) was available for analysis of proteoglycan content using standard ELISA methodology (Messner et al. 1993b). A second wash-out sample, collected at killing, was available for analysis from 69 of the knees. The discrepancy was mainly due to dry taps. 58 knees had samples available for analysis both at time zero and at killing, whereas 24 animals had samples available from both knees at both time zero and at killing. The samples collected from the experimental animals were all analyzed as one batch.

### Statistics

Based on previous experimental studies (Aroen et al. 2006), a filling difference of more than 25% was considered to be a proper level to discharge the H_01_-hypothesis of no difference in tissue filling between the patellas and the condyles. Since the experimental animals all underwent surgery in both knees, with patellar defects in one and defects of the MFC in the other, they would serve as their own control regarding tissue filling of the defects, and thus a paired Student t-test could be applied. Pre-experimental analysis to detect sample size using a power of 0.80 and a significance level of 0.05 and a standard deviation for the differences of less than 24% indicated a need for 9 animals in each group for this purpose. To evaluate the difference in tissue filling from one follow-up time point to another regarding each location separately, the need for animals was estimated to be 12 for each of the 3 follow-up time points—due to an unpaired experimental situation. Power and sample size estimations were not performed for the secondary endpoints. To cover up for possible loss of animals during follow-up, 41 rabbits were to be included in the study.

The difference in tissue filling between patellar defects and MFC defects was calculated and evaluated by one-way ANOVA and paired Student t-tests. Tissue filling with time was evaluated by one-way ANOVA and unpaired t-tests. Differences in mineralized tissue density between patellas and condyles—and the changes with time—were analyzed by one-way ANOVA and paired and unpaired t-tests. The change in proteoglycan content of synovial fluid from time zero to follow-up, named delta (…), was evaluated by ANOVA and paired t-tests. Interactions between time and location were investigated; dependent observations at the individual level were accounted for by pairwise computation of the difference between the deltas of the knees, the results being used in an ANOVA model with time to follow-up as group factor. The rise in proteoglycan content (delta) for each location at each follow-up was further analyzed by paired t-tests, the level of significance being corrected according to Bonferroni. The SPSS software package version 14 was used for statistical analysis.

## Results

### Complications

None of the animals died during surgery. Postoperatively, visual observation did not reveal any harmful effects on gait in the animals and no differences in level of activity or motion pattern between legs were observed. 5 of the 41 experimental animals died unexpectedly of unknown causes during follow-up. The knees were all unaffected, the deaths were all within 2 weeks of a follow-up time point, and the specimens were therefore kept in the study. Additionally, 4 animals had to be killed during follow-up due to impaired general health. One of these was killed 2 weeks after initial surgery and was excluded from the study. Of all the animals, complications related to the knee were observed in 14 cases: 7 knees were excluded due to patellar dislocation, 1 knee because of infection, and 6 knees due to technical failures in histology preparation.

### Macroscopic changes

Of the knees with defects but no complications, none of them had major degenerative changes at any follow-up point. Some minor degenerative changes were observed in 8 of 69 experimental knees (Table 1). There were no statistically significant differences in the number of knees with minor degenerative changes that were not related to either the location of the defect or time. Furthermore, there was no correlation between either the changes in subchondral mineralized tissue density (p = 1.0) or the changes in synovial fluid proteoglycan content (p = 0.6) and the minor degenerative changes observed.

**Table 1.** The fraction of knees with minor degenerative changes observed macroscopically at follow-up

	12 weeks	24 weeks	36 weeks
Knees with a patellar defect	3 / 8	2 / 9	1 / 17
Knees with a MFC defect	2 / 8	0 / 9	0 / 18

### Filling of the chondral defects

**Figure 6.**
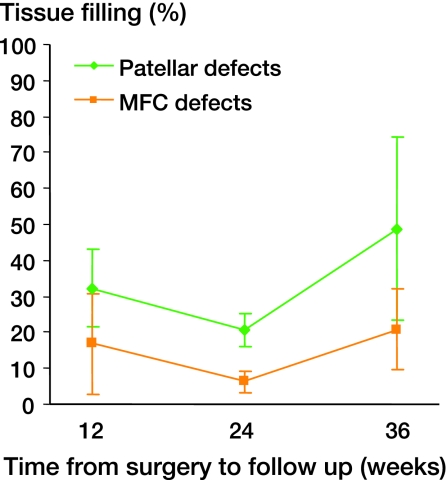
The percentage filling of the patellar and MFC chondral defects at the different time points of follow-up. For pairwise comparison, the numbers of animals at the 12-, 24-, and 36-week follow-ups were 8, 7, and 17, respectively.

There was a significantly higher degree of tissue filling in patellar defects compared to condylar defects at all follow-ups (Table 2). The difference in tissue filling between the 2 locations remained similar with time (p = 0.2). Both the patellar and condylar defects showed a change in filling with time (p = 0.02 and p = 0.02, respectively) (Figure 6), the change being apparent at the interval from 24 to 36 weeks (p = 0.003 for patellar defects and p < 0.001 for condylar defects).

**Table 2.** The percentage (SD) of tissue filling in defects. The two locations are compared pairwise at each time point of follow-up, giving the mean differences, the 95% CI, and the p-values obtained by Student t-tests

	12 weeks	24 weeks	36 weeks
	(n = 8)	(n = 7)	(n = 17)
Patellar defects	32 (11)	21 (4.5)	49 (26)
Condylar defects	17 (14)	6.2 (3.0)	21 (11)
Mean difference	15 (17)	14 (5.9)	28 (27)
95% CI of difference	1.3–30	8.8–20	14–42
p-value	0.04 **^a^**	0.001	0.001

**^a^** Not significant with Bonferroni's correction.

### Mineralized subchondral tissue

**Figure 7.**
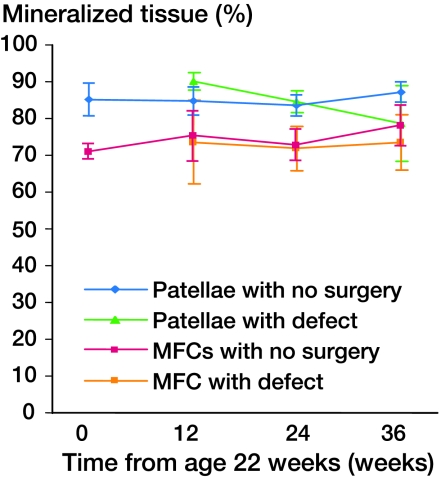
The percentage of subchondral mineralized tissue with time in patellar and MFC specimens. For pairwise comparison, the number of experimental animals at 12, 24, and 36 weeks follow-up were 8, 7, and 17, respectively. The numbers of animals with no surgery sacrificed at the 0-, 12-, 24-, and 36-week time points were 3, 3, 2, and 2, respectively.

The mineralized subchondral tissue density of the patellas with defects decreased with time (p = 0.01), whereas the density of condyles with defects remained similar with time (p = 0.9). The reduction in patellar density mainly occurred in the interval between 12 and 24 weeks (p = 0.01), contributing to a major reduction in density between 12 and 36 weeks (p < 0.001) (Figure 7). Although the descriptive statistics (95% CI) and one-way ANOVA applied on the computed pairwise differences between patellar and condylar defects showed a higher density in patellas with defects than in condyles with defects at all time points, the difference was reduced in the interval from 24 to 36 weeks (p = 0.02). These findings were supported by paired Student t-tests comparing patellar defects with condylar defects, the difference being statistically highly significant at 12 and 24 weeks, whereas the significance of the difference was borderline at 36 weeks—and not significant if Bonferroni's correction was used.

### Proteoglycan content of synovial fluid

**Figure 8.**
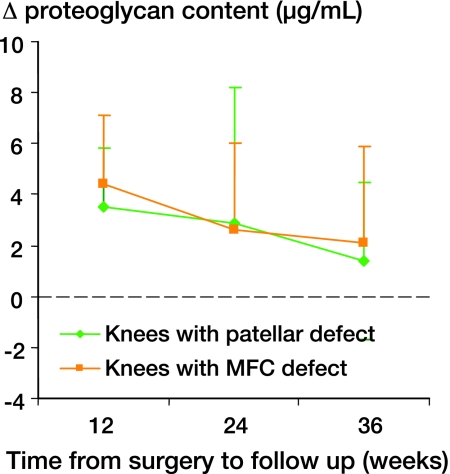
The values of change (Δ) in synovial fluid proteoglycan content from time zero to the different time points of follow up in knees with patellar and MFC defects. The number of animals at 12, 24 and 36 weeks follow up were 8, 7 and 17 respectively.

A higher proteoglycan content was detected at 12 weeks than at time zero both for knees with patellar defects (p = 0.007) and for those with condylar defects (p = 0.003), whereas the values at 24 and 36 weeks were similar to those at time zero (Figure 8). There was no interaction between defect location and time to follow-up, and there was no effect of location on the change in proteoglycan content.

## Discussion

### Effect of intraarticular location on defect filling

We found a higher degree of filling in the patellar defects than in the medial femur condylar defects at the 24- and 36-week follow-ups. At 36 weeks, the mean difference in filling was 28% with a standard deviation of 27% (p = 0.001). The large variance in spontaneous filling of ICRS grade 3b defects has been emphasized by other authors (Breinan et al. 1997). Tissue filling is to some extent assumed to be crucial in cartilage restoration of a chondral defect. However, the critical amount of tissue filling necessary to discriminate one clinical outcome from another regarding joint function, pain, disability, and reduced risk of osteoarthritis, is not well understood. According to our power and sample size estimation, a filling difference of more than 25% was considered to be a proper level to discard the H_01_-hypothesis of no difference in tissue filling between locations in the patella and the medial femoral condyle. The mean differences at 12 and 24 weeks were both below 25%. At 36 weeks, however, the mean difference was 28%; thus, the H_01_-hypothesis could be discarded. The finding indicates that the intraarticular location influences the natural history of filling of a pure chondral defect ICRS grade 3b in a long-term follow-up. To our knowledge, this issue has not been thoroughly investigated previously. The potential effect of the intraarticular location on the outcome of surgical repair of chondral defects (ICRS grade 3b) has been discussed in previous papers (Breinan et al. 1997, 2000). Breinan et al. (1997) compared autologous chondrocyte implantation (ACI) in trochlear defects to untreated controls in dogs. They did not detect any significant effect of the ACI on the amount of defect filling, in contrast to Brittberg et al. (1996) who did the same comparison of ACI and controls in patellar defects in rabbits. Breinan et al. (1997) suggested the intraarticular location of the defect (trochlea vs. patella) to be one possible explanation for the discrepancy in results. In a later study using the same dog model with 2 chondral defects (ICRS grade 3b) in trochlea, this group found a difference between proximal and distal defects in the percentage of reparative tissue that was fibrocartilage (p = 0.02), suggesting that the intraarticular location may play a role in cartilage restoration (Breinan et al. 2000). A discrepancy in results after cartilage repair, possibly related to the intraarticular location, has been noted in clinical studies also, the femoral condyle being the most favorable location (Brittberg et al. 1994, Hangody and Fules 2003, Krishnan et al. 2006).

We found an effect of the intraarticular location to be a predictive factor for the outcome of natural history of tissue filling of an ICRS grade 3b defect in the rabbit knee. We believe that knowledge of intrinsic factors that influence the outcome is essential in evaluating different aspects of cartilage restoration.

### Effect of time on defect filling

We observed a change in tissue filling of both the patellar and condylar defects with time. This difference remained similar with time, indicating that the changes in tissue filling were similar at the two locations. At both locations, the amount of filling increased from 24 to 36 weeks. This observation shows that time to follow-up is an important parameter in evaluating the results of natural history tissue filling in experimental models. The effect of time on cartilage restoration has been well studied in animal models involving defects corresponding to ICRS grade 4 (Shapiro et al. 1993, Lietman et al. 2002). On the other hand, the natural time course of ICRS grade 3 defects is less well known. In their untreated control defects, Grande et al. (1989) observed 17% spontaneous filling of a 3-mm “full-thickness” defect (through all chondral layers into the calcified zone, corresponding to ICRS grade 3c) in the rabbit patella at 6 weeks of follow-up. Using the same rabbit model as Grande, Brittberg et al. (1996) increased the follow-up time and reported 29% spontaneous filling at 12 weeks, which was similar to the 32% filling of patellar defects obtained at 12 weeks in our study. However, in our study the amount of tissue filling increased to 49% at the 36-week follow-up. The increase in spontaneous tissue filling after 12 weeks is in line with studies using other animal models. Breinan et al. (1998) reported 35% spontaneous filling of a 4-mm defect corresponding to ICRS grade 3b in the knee trochlea of dogs at 3 months. In a separate study using the same dog model, these authors obtained 41% spontaneous filling at 12 months, which increased to 76% at 18 months (Breinan et al. 1997).

In our study, for both locations the amount of filling tended to decline from 12 to 24 weeks and then increased from 24 to 36 weeks. This observation is probably not in conflict with those of Frisbie et al. (1999) investigating 1 cm^2^ “full thickness” defects (removing all the calcified cartilage, but preserving the bone plate; corresponding to ICRS grade 3c) in a horse model. Due to merging of the data from the groups in various ways, the results presented are not easy to interpret, but the mean filling of the treated defects and the control defects together was 44% at 4 months and 54% at 12 months; the filling of the controls when the 2 time points were merged together was lower than the filling of the treated defects. In another study using the same model, the same authors reported 52% filling of control defects at 8 weeks of follow-up (Frisbie et al. 2003). The combined results from these 2 studies seem to give the same natural history for filling of a defect as we found: there was a high percentage of filling at an early follow-up, with a tendency towards decrease at a medium-time follow-up, and increasing again at long-term follow-up. We observed such a time course for both locations, but we cannot explain the phenomenon. We believe, however, that knowledge of the natural time course in a given model is essential for evaluation of different aspects of cartilage restoration.

### Mineralized subchondral tissue density

In contrast to the density of the subchondral tissue in the condyles, the density of the mineralized subchondral tissue in the patellas changed with time. This indicates that the intraarticular location is also a predictive factor for subchondral bone changes related to chondral defects of ICRS grade 3b in the rabbit knee. To our knowledge, this issue has not been discussed in any of the previous literature.

One weakness of our study was the lack of sufficient numbers of control patellas and condyles for evaluation of subchondral bone changes. Our intention was to use patellas from knees with condylar defects, and femoral condyles from knees with patellar defects as controls. Additional animals were added only to make up a sufficient number of control specimens. We learned, however, that there is a potential effect of chronic cartilage lesions in one articular surface to cause changes to the subchondral bone of other articular surfaces within the same joint (Marijnissen et al. 2002, Sniekers et al. 2008). The contralateral patella/condyle of the experimental animals was therefore excluded, leaving us with a number of “controls” that was too small for statistical analysis. However, the observation that the density of mineralized tissue in patellas changed with time—whereas in condyles it did not—may be an important finding. Subchondral bone changes are associated with the initiation and progression of osteoarthritis (OA) (Radin and Rose 1986, Burr 1998, 2004). There are, however, no objective criteria regarding the degree of changes that would indicate a substantial initiation or progression of OA. Thus, no estimations of power or sample size were performed considering the changes in the density of subchondral mineralized tissue. Whether the statistically significant changes we found have any clinically implications thus remains uncertain.

Some authors have emphasized that changes in the calcified cartilage, being part of the mineralized subchondral tissue, may play a role in the pathogenesis of OA (Burr 2004). Thus, defects involving iatrogen damage to the calcified cartilage (ICRS grade 3c) should probably be distinguished from pure chondral lesions (ICRS grade 3b) in evaluating the natural history of the subchondral mineralized tissue. The impairment of a well-defined pure chondral defect (ICRS grade 3b) on the subchondral bone is less well known. By merging data from 12 and 18 months of follow-up in dogs, Breinan et al. (1997) reported resorption of the subchondral bone leading to moderate to severe bone loss in 3 of 14 untreated ICRS grade 3b defects in the trochlea. They offered no explanation for the changes, but raised the possibility of having caused damage to the calcified cartilage during surgery, made evident by studying fresh defects in cadaver dogs. To reduce the risk of damage to the tidemark and the underlying calcified cartilage in our study, we used a stereo microscope and small instruments in preparing the defect.

We found a reduction in the density of mineralized subchondral tissue from 12 to 36 weeks in the patellas with defects. The time course is in agreement with that of changes in corresponding parameters reported in experimental OA models. In their model of OA in dogs, Sniekers et al. (2008) reported a 6% increase in bone volume fraction at ten weeks, which subsided to a 13% loss at 20 weeks. The finding of reduced bone volume fraction combined with thinning of the subchondral bone plate as well in early degenerative joint disease is also in agreement with other studies (Dedrick et al. 1993).

Although the filling of the defects in the condyles was lower at all time points, the mineralized subchondral tissue in the condyles seemed to tolerate the defect better, showing no detectable changes in density with time. Thus, the intraarticular location may be a predictive factor for subchondral bone changes related to chondral defects of ICRS grade 3b in the rabbit knee.

### Proteoglycan content of synovial fluid

A rise in synovial fluid proteoglycan content was detected in the knees of animals killed 12 weeks after surgery. There was no statistically significant difference between knees with patellar defects and knees with condylar defects. The rise observed at 12 weeks subsided with time, being similar to time-zero values at 24 and 36 weeks of follow-up.

Increased concentrations of proteoglycan fragments in the joint fluid have been found to be associated with trauma and surgery in humans (Lohmander et al. 1989, Odenbring et al. 1991), and with increasing knee OA in rabbits (Messner et al. 1993a). In their rabbit OA model, Messner et al. found an increase at 3 months which they explained by the effect of surgery, the values decreasing to normal at 6 months and then increasing again at 12 months, which they explained as possibly being related to initial degeneration. These findings are not in conflict with the observations in our study; there was a rise in proteoglycan content at 12 weeks, subsiding to values similar to time zero at the 24- and 36-week follow-ups. Consequently, the proteoglycan concentrations of synovial fluid in our study did not indicate a degenerative process during the observation period.

### Weaknesses of the study

The sample size estimation indicated a need for 12 animals for each of the 3 follow-ups. A sufficient number was obtained at 36 weeks of follow-up, whereas the numbers at 12 and 24 weeks were below that due to a high number of complications and failures in histological preparation. The data obtained at 36 weeks thus represent the most valuable information. Complications and loss of animals related to the rabbit model have also been described by other authors (Brittberg et al. 1996).

### Strengths of the study

The importance of defining the depth of experimentally produced defects in relation to the different layers of the joint organ—and evaluation of the results in relation to that—has been emphasized by others (Brittberg et al. 1996, Breinan et al. 1997, Frisbie et al. 1999, 2003, Hunziker 1999, Burr 2004). Even the bias of not removing all the tissue of a layer as intended, or causing damage to the layer beneath, has been a topic in discussing the results of cartilage restoration (Breinan et al. 1997, Frisbie et al. 1999). Methodologically, our study was strengthened by the use of a stereo microscope, which ensured removal of all the cartilage without causing damage to the calcified layer and the tissue beneath in preparing the defects. In addition, we used the most commonly applied animal model in cartilage repair.
